# Loss of Genetic Diversity among Ocelots in the United States during the 20^th^ Century Linked to Human Induced Population Reductions

**DOI:** 10.1371/journal.pone.0089384

**Published:** 2014-02-26

**Authors:** Jan E. Janecka, Michael E. Tewes, Linda Laack, Arturo Caso, Lon I. Grassman, Rodney L. Honeycutt

**Affiliations:** 1 Caesar Kleberg Wildlife Research Institute, Texas A & M University-Kingsville, Kingsville, Texas, United States of America; 2 Laguna Atascosa National Wildlife Refuge, Rio Hondo, Texas, United States of America; 3 Natural Science Division, Pepperdine University, Malibu, California, United States of America; Institute of Evolutionary Biology (CSIC-UPF), Spain

## Abstract

Ocelots (*Leopardus pardalis*) in the United States currently exhibit low levels of genetic diversity. One hypothesis for this observation is that habitat fragmentation, resulting from human induced changes in the landscape during the 20^th^ century, created island populations with highly reduced gene flow and increased genetic drift and inbreeding. In an effort to investigate this, we used a portion of the mitochondrial control region and 11 autosomal microsatellite loci to examine historical levels of genetic diversity and infer temporal changes in ocelot populations between 1853 and 2005. Levels of genetic diversity were higher in historical ocelot populations than in extant populations from Texas. The earliest documented loss of mitochondrial haplotype diversity occurred at Laguna Atascosa National Wildlife Refuge. The second extant population inhabiting private lands in Willacy County retained higher levels of genetic diversity through the 1990s, but subsequently lost diversity over the next decade. A similar pattern was observed for autosomal microsatellite loci. This supports the argument that low levels of genetic diversity in Texas are related to human induced population reductions and fragmentation, both of which threaten the remaining ocelots in the United States. At this time, the best means of mitigating the continued erosion of genetic variation are translocation of individuals either from larger populations in Mexico to Texas, or between the Texas populations.

## Introduction

The ocelot (*Leopardus pardalis*) is a small (<15 kilogram), solitary, Neotropical felid [1]. Although common throughout Central and South America, it is currently endangered in the United States [1]–[3]. Historically, the ocelot’s distribution included Texas, Arkansas, Louisiana, and Arizona [3]. It was extirpated from the vast majority of its range in the United States during the 20^th^ century, and by the 1960s the distribution became restricted to southern Texas [1], [3], [4]. Throughout the 1970s and 1980s, the remaining populations of ocelot in Texas continued to experience declines. Currently, the last known breeding populations within the United States are predominantly confined to small habitat patches separated by ≈30 km of extensive croplands and more open rangeland [5]–[14]. Data from both telemetry and genetic analyses have confirmed that the human modified landscape separating these two populations represents a virtually impenetrable barrier to dispersal [9]–[15].

The two known ocelot populations in the United States are characterized by small effective population size (N_e_<14), high genetic drift (F_st_ = 0.163, *P*<0.001), and reduced genetic variation (H_e_ = 0.399 in Laguna Atascosa National Wildlife Refuge [LANWR] and He = 0.553 in Willacy County) [11], [12]. This may be the result of human activities in the early 1900s including uncontrolled harvesting of ocelots and extensive thornshrub habitat removal [3]. Information regarding the role that anthropogenic factors played in the loss of genetic diversity in ocelot populations in the United States has important implications for ocelot recovery efforts currently underway. For habitat specialists like the ocelot, fragmentation via anthropogenic perturbations disrupts connectivity between populations, increases human caused mortality, and contributes to demographic instability [16]–[18]. This also decreases genetic diversity and increases divergence as a consequence of drift and inbreeding [19]. Deleterious effects include loss of adaptive variation and an increase in the frequency of detrimental alleles [20], [21]; both of these factors can lead to inbreeding depression [20]–[24].

The effects of inbreeding depression, loss of adaptive diversity, and demographic instability greatly increase extinction risks of small, fragmented populations [25]–[31]. Indeed, there are cases where rapid loss of genetic variation has contributed to local extinctions [21], [26], [27], [32]. The impact of inbreeding and drift depends largely on both the length of time the population has remained small and the biology of the species. These processes also predictably affect neutral genetic variation [19], [33]. Therefore, temporal changes at neutral loci provide a historical perspective on the degree to which reductions in population size and gene flow have impacted the overall viability of currently declining species. In fact, there are strong correlations between neutral diversity and adaptive variation, inbreeding depression, and extinction risks [34]–[38].

If isolated populations of ocelot reveal evidence of a recent and continued decline in genetic diversity, then this will influence the decision-making process used in recovery planning. For instance, under such a scenario, maintaining current ocelot habitat may not be enough to offset the demographic and genetic consequences of small effective population size, isolation, loss of adaptive variation, and inbreeding. Translocations may be required to mitigate the impact of inbreeding depression and reestablish metapopulation connectivity, thus increasing the likelihood of ocelots persisting until additional habitat either becomes available or is restored.

We therefore explored temporal change in genetic diversity by examining historical (1853–1956) and extant ocelot populations (1984–2005) occupying the Tamaulipan Biotic Province (TBP) of southern Texas and northeastern Mexico. A portion of the mitochondrial DNA (mtDNA) control region and 11 nuclear microsatellite loci were used to address the following questions: (1) What were historical levels of genetic variation in ocelot populations in the United States? (2) Have levels of genetic variation changed in these populations over the last 100 years?

## Materials and Methods

### Sample Collection

**Figure 1:**
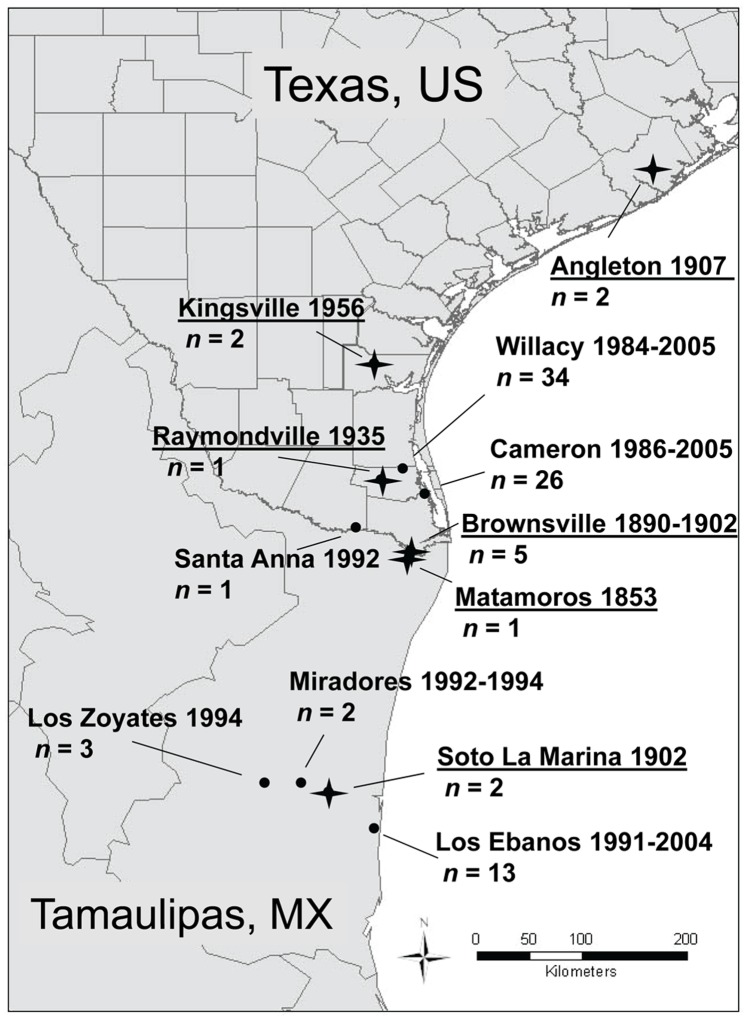
Map showing primary localities examined during this study, the dates samples were collected, and the number of samples. Contemporary populations are identified with solid dots and the localities where museum specimens originated are underlined and marked with a star.

Museum samples (n = 15) used in this study were generously provided by the National Museum of Natural History (NMNH) of the Smithsonian Institution. Museum accession numbers and information available on the specimens sampled are provided in the Table S1. The historical samples were from the following locations: (1) Brownsville, Texas (1890–1892, n = 5, A0044601, A0044604, A0044605, A46491, and A0046119); (2) Angleton, Texas (1907, n = 2, 150363 and 150364); (3) Raymondville, Texas (1935, n = 1, 251587); (4) Kingsville, Texas (1956, n = 2, 287771 and 287774); (5) Matamoros, Tamaulipas, Mexico (1853, n = 1, A1364); (6) Perez, Veracruz, Mexico (1893, n = 2, 132522 and 132524); and (7) Soto la Marina, Tamaulipas, Mexico (1902, n = 2, 125716 and 125717) (Fig. 1).

A subset of mitochondrial and microsatellite data from Janecka et al. [11], [12] was used to compare genetic diversity of the above historical samples to contemporary ocelot populations in Texas and northeastern Mexico (Table S1). This included control region sequences and genotypes for extant ocelots (n = 101) occurring in the TBP, which is a combination of Nearctic and Neotropical fauna and flora [39]. These ocelots were originally sampled during telemetry studies conducted between 1984 and 2005 [5], [8], [13]–[15], [40], [41] in the following study sites: (1) Laguna Atascosa National Wildlife Refuge (LANWR), Cameron County, Texas (Cameron, n* = *40); (2) private ranches in northern Willacy County, Texas (Willacy, n* = *34); and (3) Los Ebanos Ranch, Mexico (Mexico, n = 14, Fig. 1). Thirteen ocelots originated from outside of the primary populations. These included the following areas in Texas: (1) Santa Anna National Wildlife Refuge (SANWR), Hidalgo County (live-capture 1992, n = 1); (2) Port Brownsville, Cameron County (live-capture 1998, n = 1); (3) Port Mansfield (road kills 1993 and 2004, n = 2); (4) Lyford, Willacy County (road kill 1996, n = 1); (5) Sarita, Kenedy County (road kills 1990 and 1997, n = 2); and (6) Highway 186, Willacy County (road kill 1999, n = 1). Five ocelots were from two areas near Abasolo, Tamaulipas, Mexico: (1) Miradores (live-capture 1992 and 1994, n = 2) and (2) Los Zoyates (live-capture 1994, n = 3).

### Ethics Statement

There were no live animals trapped or handled during this study. All samples from live animals represented archival material collected by either Texas A&M University-Kingsville or Laguna Atascosa National Wildlife Refuge during previous research projects. Please refer to the original publications to obtain information on the trapping and handling of ocelots [8], [13]–[15], [40], [41].

### DNA Extraction

Total DNA was extracted from museum specimens using a PureGene DNA extraction kit (Gentra Systems, Minneapolis, Minnesota, United States). Ten to 20 mg of crushed bone or diced skin was placed in 600 µl of Cell Lysis Solution, 3 µl of Proteinase K (20 mg/ml) was added, and the tubes were inverted 25X. Samples were incubated at 55°C for 3 days with the addition of 1.5 µl of Proteinase K every 24 hrs. Tubes were periodically inverted. After incubation, 3 µl of RNase Solution (4 mg/ml) was added, tubes were inverted 25X, and incubated for 15 min at 37°C. Samples were cooled to 4°C for 10 min and 200 µl Protein Precipitation Solution was added. Tubes were then vortexed for 20 sec and centrifuged at 16,000 g for 5 min. The supernatant was pipetted into 600 µl of 100% isopropanol with 1 µl of glycogen. Tubes were inverted 50X and centrifuged at 16,000 g for 3 min to pellet the DNA. The isopropanol was decanted and tubes were inverted on clean absorbent filter paper. The DNA pellet was washed with 600 µl of 70% ethanol, inverted 3X, and centrifuged at 16,000 g for 1 min. Ethanol was decanted, tubes were inverted on clean absorbent filter paper, and air dried for 20 min. The DNA pellet was hydrated in 40 µl of DNA Hydration Solution at 65°C for 1 h and stored at 4°C.

### Precautions Taken with Museum Samples

All DNA extractions and PCR preparations of museum samples were conducted in a lab dedicated to genetic analyses of museum samples and physically separated from areas where work with modern DNA was performed. There were no contemporary samples of any species present in the museum lab. The equipment used for the museum samples, including pipetmen, centrifuge, refrigerator, freezer, tube racks, and heat blocks was either new, or decontaminated and subsequently used solely for museum work. Prior to each extraction or PCR preparation, all surfaces, bench tops, and equipment were cleaned with 20% bleach. Irradiation from a UV hood lamp was then used to destroy any remaining potential DNA contaminants on tubes, tips, racks, and pipetmen. Personnel working with ancient DNA wore clothes and shoes not previously worn in a laboratory where modern DNA was handled. Aerosol tips were used for all extractions and PCR preparations, which were performed in a UV sterilized hood. Negative controls were included with every extraction and monitored to ensure that no trace contamination was present.

We did not repeat the sequencing or genotyping of museum samples; however, the following evidence suggests that we had data of sufficient quality for analysis: (1) We did not observe any contamination in negative controls. (2) The two new haplotypes observed in museum samples were never previously detected in contemporary samples and were sequenced from both strands. (3) We detected microsatellite alleles in museum samples that were not previously observed in contemporary samples. (4) We did not observe any genotypes with more than 2 alleles, which would have suggested cross-contamination. (5) For low quality DNA samples the most common errors are PCR failure and allele drop out, both of which would reduce diversity observed in museum samples; therefore, our study provides a conservative estimate of historical ocelot variation.

### MtDNA Sequencing

Mitochondrial sequence data for museum specimens were generated during this study (Table S1). Sequence data for contemporary samples were from Janecka et al. [11]. A 436-base pair (bp) fragment of the mtDNA control region was amplified using the polymerase chain reaction (PCR) and primers CCR-F and CCR-R [42] modified to complement the ocelot sequence (CCR-OCELOT-F: 5′CTCAACTATCCGAAAGAGCTT; CCR-OCELOT-R: 5′CCTGTGGAACATTAGGAATT) [43]. After the sequences were trimmed of primers and low quality bases, the remaining 418-bp segment spanned positions 16,811 to 17,009 and 1 to 226 of the domestic cat (*Felis silvestris catus*) mitochondrial genome FCU20753. This is located in the central conserved region between repetitive sequences I and II [42], [44].

The precautions taken to ensure that all sequences obtained from museum samples were not contaminants are described in the section above. We performed PCR amplification in 10 µl volumes containing 0.2 mM of each dNTP, 1X PCR HotMaster *Taq* buffer (Eppendorf, Hamburg, Germany), 0.25 units of HotMaster *Taq*, 0.25 µM of forward primer, 0.25 µM of reverse primer, 0.1 µg/1 µl bovine serum albumin (New England Biolabs, Ipswich, Massachusetts, United States), and 10–20 ng DNA template. Reaction conditions included an initial denaturing step of 94°C for 1 min followed by 50 cycles of 94°C for 15 sec, 54°C for 30 sec, 72°C for 60 sec, and a final extension step of 72°C for 2 min. PCR products were sequenced using an ABI BigDye v. 1.1 Terminator Kit (Applied Biosystems, Foster City, California, United States) and an ABI 3100 automated sequencer by the Laboratory of Plant Genomics and Technology or an ABI3730 in the Molecular Cytogenetics and Genomics Laboratory, Texas A&M University. Sequences were obtained in both directions, then aligned and edited using SEQUENCHER v. 3.0 (Gene Codes Corporation, Ann Arbor, Michigan, United States).

### Microsatellite Genotyping

Eleven microsatellite loci (FCA008, FCA023, FCA035, FCA043, FCA045, FCA077, FCA082, FCA096, FCA105, FCA117, and FCA126) previously isolated and mapped in the domestic cat were used [45]. In this study, genotyping data were generated for museum specimens and three additional loci (FCA043, FCA096 and FCA105) for contemporary Mexico samples (Table S1). Remaining microsatellite genotypes were from Janecka et al. [11], [12]. PCR reactions were conducted in 10 µl reaction volumes containing 0.2 mM of each dNTP, 1X PCR HotMaster *Taq* buffer, 0.25 units of HotMaster *Taq*, 0.24 µM forward primer, 0.24 µM reverse primer, 0.1 µg/µl bovine serum albumin, and 5–20 ng DNA template. Conditions for PCR amplification included an initial denaturing step of 94°C for 1 min, 50 cycles of 94°C for 30 sec, 53°C for 30 sec, 72°C for 60 sec, and a final extension step of 72°C for 2 min. PCR products were visualized on a 1.2% agarose gel stained with ethidium bromide (0.5 µg/ml) and then exposed to ultraviolet light. Samples with visible PCR product were genotyped with an ABI 3100 automated sequencer in the Laboratory of Plant Genomics and Technology and sized using GENOTYPER v. 2.0 (Applied Biosystems).

### MtDNA Data Analysis

Sequence alignments were performed in CLUSTAL-X [46], and population statistics including the number of variable sites, haplotype diversity (H), nucleotide diversity (π) and mean number of nucleotide differences were calculated in DNASP v. 4.10.8 [47]. A minimum spanning network of haplotypes was constructed in ARLEQUIN v. 3.0 [48] and plotted to represent relationships among haplotypes. Tests for departure of haplotype frequencies from neutrality were performed using Tajima’s D test and Fu and Li’s D test in DNASP [49], [50].

Two methods were used to compare mtDNA variation in ocelot populations. An exact test for population differentiation based on haplotype frequencies was implemented in ARLEQUIN. Population structure was tested using pairwise F_st_ estimates in ARLEQUIN. Estimates of F_st_ were tested for significance against the null distribution of F_st_ values obtained from 1,000 permutations of haplotypes under the null model of panmixia in ARLEQUIN.

### Microsatellite Data Analysis

Measures of genetic variability, including expected heterozygosity (H_e_), observed heterozygosity (H_o_), number of alleles (A_N_), and number of private alleles (A_P1_) were estimated using GENALEX v. 6.0 [51]. GENEPOP v. 3.1 [52] was used to test for linkage disequilibrium and Hardy-Weinberg equilibrium (HWE). Populations were tested for deviations from equilibrium at each locus and across all loci in GENALEX. Bonferroni corrections were applied to comparisons involving multiple tests for HWE and linkage disequilibrium [53]. Allelic richness (A_R1_) was estimated in FSTAT v. 2.3.9 and using the rarefaction approach (A_R2_) in HP-RARE v. 1.1 [54], [55]. Private alleles (A_P2_) were also estimated using the rarefaction approach in HP-RARE to take into account different sample sizes. Pairwise F_st_ between populations was estimated using AMOVA analysis and tested for significance with 9,999 permutations in GENALEX.

## Results

### MtDNA

**Figure 2:**
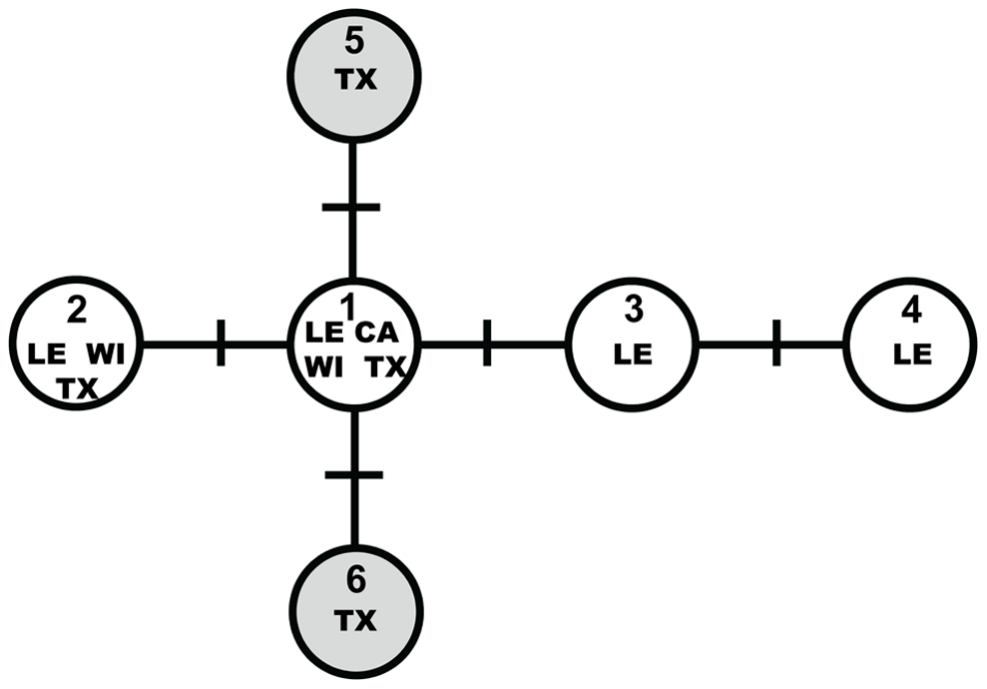
The minimum spanning network representing the most parsimonious mutation pathway between 6 ocelot haplotypes observed in Texas and northeastern Mexico. Each hatch mark represents a single nucleotide point mutation. The populations in which haplotypes were observed are noted; CA = Cameron, WI = Willacy, LE = Los Ebanos, Mexico, TX = historical Texas samples. Haplotypes represented with shaded circles were observed only in museum samples originating in Texas prior to 1956.

A 418-bp fragment of the control region was sequenced and aligned for 86 ocelots from extant populations and 15 museum specimens from the Tamaulipan Biotic Province. There were 5 variable sites distributed among 6 haplotypes, and each haplotype differed by a single mutation (Table 1, Fig. 2). Two of these (hap 5 and hap 6) were novel and only detected in museum specimens (GenBank Accessions KF746960 and KF746961). The central haplotype was found in all populations and at the highest frequencies (Tables 1 and 2, Fig. 2). Only 1 haplotype was observed in the Cameron population (hap 1) between 1986 and 2004. Two haplotypes (hap 1 and hap 2) were identified in Willacy 1984–2005, the most common of which was the haplotype observed in Cameron. Hap 1 was found in 7 of the extant Texas ocelots originating outside the primary populations and hap 2 in a road-killed ocelot found 9.6 km west of Port Mansfield in 2004. Two additional haplotypes (hap 3 and hap 4) were observed in Los Ebanos 1991–2004, and 2 more (hap 5 and hap 6) in the historical Texas samples originating between 1853 and 1956 (Table 2, Fig. 2). Hap 1 was also observed in the two historical ocelot specimens from Perez (1903) and the two from Soto La Marina (1902).

**Table 1:** Mitochondrial control region diversity of ocelots in Texas and Mexico.

Location	Date	N^a^	VS^b^	Haplotypes	H^c^	SD^d^	π^e^	SD
All Ocelots	1853–2005	101	5	hap 1, 2, 3, 4, 5, 6	0.426	0.084	0.00127	0.00030
**Contemporary Ocelots Sampled**								
Texas/Mexico	1986–2005	86	3	hap 1, 2, 3, 4	0.254	0.060	0.00077	0.00020
Texas - Combined	1986–2005	68	1	hap 1, 2	0.163	0.057	0.00039	0.00014
Cameron - Combined	1986–2005	26	0	hap 1	0	0	0	0
Cameron	1986–1989	11	0	hap 1	0	0	0	0
	1996–1998	10	0	hap 1	0	0	0	0
	2004–2005	5	0	hap 1	0	0	0	0
Willacy - Combined	1984–2005	34	1	hap 1, 2	0.258	0.009	0.00062	0.00021
Willacy	1984–1990	8	1	hap 1, 2	0.536	0.123	0.00128	0.00029
	1994–1998	16	1	hap 1, 2	0.233	0.126	0.00056	0.00023
	2005	10	0	hap 1	0	0	0	0
Other areas in Texas^f^	1990–2004	8	1	hap 1,2	0.250	0.180	0.00060	0.00043
Mexico - Los Ebanos	1991–1998	10	3	hap 1, 2, 3, 4	0.733	0.012	0.00282	0.00066
	2001–2004	3	1	hap 1, 2	0.667	0.314	0.00319	0.00015
Other areas in Mexico^g^	1992–1994	5	1	hap 1	0	0	0	0
**Historical Ocelots Sampled**								
Texas/Mexico	1853–1956	15	3	hap 1, 2, 5, 6	0.543	0.133	0.00146	0.00043
Texas	1853–1956	11	3	hap 1, 2, 5, 6	0.673	0.123	0.00191	0.00049
Mexico	1902–1903	4	0	hap 1	0	0	0	0

Number of variable sites, haplotypes, haplotype diversity, and nucleotide diversity for a 418-bp segment of the mitochondrial control region for 3 contemporary ocelot populations and historical ocelots sampled with museum specimens.

a N = Number of samples.

b VS = Variable sites.

c H = Haplotype diversity.

d SD = Standard deviation.

e π = Nucleotide diversity.

f Areas sampled outside of the two primary Texas populations including Santa Anna National Wildlife Refuge, Port Brownsville, Port Mansfield, Lyford, Sarita, and Highway 186 in Willacy County.

g Areas sampled outside of the primary Mexico population including Los Zoyates and Miradores.

**Table 2:** MtDNA haplotype frequencies in ocelot populations.

	Relative Frequency				
	Contemporary			Historical	
Haplotype	Cameron	Willacy	Los Ebanos	Mexico	Texas
1	1.000	0.846	0.500	1.000	0.545
2	0	0.154	0.100	0	0.273
3	0	0	0.200	0	0
4	0	0	0.200	0	0
5	0	0	0	0	0.091
6	0	0	0	0	0.091

Haplotype frequencies in contemporary (1984–2005) and historical ocelot populations (Mexico 1902–1903 and Texas 1853–1956). Cameron and Willacy are counties in Texas, and Los Ebanos is in southern Tamaulipas, Mexico.

To compare genetic diversity of contemporary and historical ocelot populations in Texas, we used 10 museum samples collected in Texas and 1 ocelot museum specimen from Matamoros (Figure 1). Brownsville and Matamoros are cities on either side of the Rio Grande and represent the same area. Therefore, the specimen from Matamoros was included among the historical Texas samples. The highest levels of haplotype and nucleotide diversity were observed in Los Ebanos (H = 0.733, *SD* = 0.012; π = 0.00282, *SD* = 0.00066) sampled between 1991 and 1998 (Table 1). Levels of genetic diversity in historical Texas ocelots 1853–1956 (H = 0.673, *SD* = 0.123; π = 0.00191, *SD* = 0.00049) were comparable to those from the extant Los Ebanos population. In Willacy, haplotype diversity decreased from H = 0.536 (*SD* = 0.123) in 1984–1990, to H = 0.233 (*SD* = 0.126) in 1994–1998, and to H = 0 in 2005 (Table 1). Nucleotide diversity in Willacy was π = 0.00128 (*SD* = 0.00029) in 1984–1990 and decreased to π = 0.00056 (*SD* = 0.00023) in 1994–1998 and to π = 0 in 2005 (Table 1).

Estimates of haplotype diversity in Los Ebanos, historical Texas samples, and Willacy 1984–1990 were not significantly different. However, haplotype diversity of the Willacy population in 1994–1998 was significantly lower than in Los Ebanos (*P*<0.05). Nucleotide diversity of the Los Ebanos population was also significantly higher than the other populations sampled (*P*<0.05). The historical samples from Texas had higher nucleotide diversity than the contemporary Willacy and Cameron populations.

The greatest level of genetic differentiation was between the Cameron 1996–1998 and Los Ebanos 1991–1998 populations with F_st_ = 0.159 (*P = *0.028) (Table 3). The lowest level of differentiation was between the Willacy 1994–1998 and historical Texas samples 1853–1956 with F_st_ = −0.025 (*P* = 0.357). Haplotype frequencies of the historical Texas samples were most similar to the Willacy population and most divergent from the Cameron population.

**Table 3:** Levels of mtDNA population differentiation among ocelots.

	Cameron	Willacy	Los Ebanos,Mexico	TexasHistorical
Cameron	–	0.485	0.028***	0.857
Willacy	0.055	–	0.065	0.357
Los Ebanos,Mexico	0.159	0.060	–	0.229
Texas Historical	0.108	−0.025	0.039	–

Estimates of population differentiation derived from mtDNA control region. The bottom left of the matrix shows pairwise F_st_ estimates. The top right of the matrix shows *P*-values for the exact test for genetic differentiation using haplotype frequencies. Significant values are noted with asterisks (*i.e.* ***).

### Microsatellite Data

Eleven microsatellite loci were genotyped in the historical specimens and compared to contemporary samples. The microsatellite data set was archived in DRYAD. However, not all loci were successfully genotyped in every museum sample; therefore, data from only a mean of 5 samples per locus were obtained. Genotypes for the same 11 loci were also obtained for a subset of ocelots from contemporary populations (Table 4). After Bonferroni correction, all loci were in HWE and showed no linkage disequilibrium. Two loci were monomorphic in the Cameron population (FCA043 and FCA096). All loci were polymorphic in the other two extant populations and in historical samples.

**Table 4:** Microsatellite variation among ocelots from Texas and Mexico.

Locality	Date Sampled	N^a^	P^b^	H_e_ c	SE^d^	A_N_ e	SE	A_R1_ f	A_R2_ g	A_P1_ h	A_P2_ i
All Samples	1890–2005	68	1.00	0.607	0.050	6.36	0.56	2.97		n/a	n/a
Cameron	1996–2005	26	0.82	0.389	0.078	2.46	0.37	1.82	2.24	0	0.10
Willacy	1996–2005	23	1.00	0.561	0.042	3.18	0.33	2.35	2.86	3	0.26
Los Ebanos	1994–1998	10	1.00	0.634	0.058	4.36	0.36	3.08	3.85	15	1.26
Texas Historical	1890–1935	9^j^	1.00	0.642	0.034	3.82	0.33	3.13	3.70	11	1.06

Measures of genetic variation of 11 microsatellite loci among contemporary ocelots sampled in Texas (Willacy and Cameron), northeastern Mexico (Los Ebanos) and ocelot museum specimens collected in the region (Texas Historical).

a N = Sample size.

b P = Polymorphic loci.

c H_e_ = Expected heterozygosity.

d SE = Standard error.

e A_N_ = Number of alleles.

f A_R1_ =  Allelic richness estimated in FSTAT.

g A_R2_ =  Allelic richness estimated using rarefaction to account for samples size differences in HP-RARE.

h A_P1_ =  Private alleles.

i A_P2_ =  Private alleles estimated using rarefaction to account for samples size differences in HP-RARE.

j Due to PCR failure the mean number samples per locus in museum specimens for which genotypes were obtained was 5.

Mean number of alleles was lowest in Cameron (A_N_ *= *2.46, *SE* = 0.37) and highest in Los Ebanos (A_N_ = 4.36, *SE* = 0.36; Tables 4 and 5). Number of alleles in the historical Texas samples (A* = *3.82, *SE* = 0.33) were comparable to Los Ebanos. Mean number of alleles was not significantly different between Cameron and Willacy; however, both were significantly (*P*<0.05) different from Los Ebanos and the historical Texas samples. The number of alleles was not significantly different between Los Ebanos and historical Texas samples. Allelic richness estimated directly from the population samples and using the rarefaction method in Texas was highest in the historical population and lowest in Cameron (Table 4). There were no private alleles in the Cameron population, but there were 3 in Willacy (Table 4). In contrast, 11 alleles in the historical samples from Texas were not observed in any contemporary population. The greatest number of private alleles, 15, was observed in Mexico (Table 4). The estimated private alleles based on rarefaction were also lowest in the two contemporary Texas populations (Table 4).

**Table 5:** Genetic diversity of individual microsatellite loci in ocelots from Texas and Mexico.

	Cameron, Texas					Willacy, Texas					Los Ebanos, Mexico					Texas Historical (1853–1935)				
Locus	N^a^	A_N_ b	H_o_ c	H_e_ d	F_IS_ e	N	A_N_	H_o_	H_e_	F_is_	N	A_N_	H_o_	H_e_	F_is_	N	A_N_	H_o_	H_e_	F_is_
FCA008	26	4	0.73	0.73	−0.01	23	4	0.83	0.71	−0.16	10	4	0.80	0.67	−0.19	5	3	0.40	0.58	0.31
FCA023	26	2	0.27	0.33	0.19	23	2	0.57	0.47	−0.21	10	2	0.10	0.10	−0.05	5	4	0.60	0.66	0.09
FCA035	26	3	0.65	0.55	−0.19	23	4	0.83	0.65	−0.28	10	6	0.80	0.80	0.00	5	5	0.40	0.74	0.46
FCA043	26	1	0.00	0.00	n/a	23	2	0.43	0.42	−0.03	9	5	0.56	0.59	0.05	9	4	0.22	0.44	0.50
FCA045	25	2	0.48	0.40	−0.19	23	3	0.83	0.64	−0.30	9	4	0.56	0.70	0.20	5	4	0.20	0.74	0.73
FCA077	26	2	0.27	0.23	−0.16	23	3	0.43	0.55	0.21	10	5	0.70	0.71	0.01	3	4	0.67	0.72	0.08
FCA082	26	3	0.54	0.57	0.05	23	4	0.83	0.66	−0.26	9	4	1.00	0.66	−0.51	3	2	0.33	0.50	0.33
FCA096	26	1	0.00	0.00	n/a	23	2	0.26	0.29	0.09	10	3	0.50	0.59	0.15	5	3	0.80	0.54	−0.48
FCA105	26	2	0.27	0.23	−0.16	22	2	0.64	0.50	−0.28	10	6	0.60	0.78	0.23	3	4	0.67	0.72	0.08
FCA117	25	2	0.52	0.50	−0.05	23	4	0.61	0.54	−0.14	10	4	0.40	0.70	0.43	5	3	0.40	0.62	0.35
FCA126	26	5	0.88	0.74	−0.19	23	5	0.87	0.76	−0.14	10	5	0.90	0.70	−0.29	7	6	0.71	0.80	0.10
Mean	26	2.5	0.42	0.39	−0.08	23	3.2	0.65	0.56	−0.14	10	4.4	0.63	0.63	0.00	5	3.8	0.49	0.64	0.23
*SE*	0.1	0.4	0.08	0.08	0.04	0.1	0.8	0.06	0.04	0.05	0.1	0.3	0.08	0.06	0.08	0.5	0.3	0.06	0.03	0.10

Genetic diversity estimates for microsatellites genotyped in two contemporary ocelot Texas populations, one contemporary Mexico population, and historical ocelot samples.

a N = Number of individuals genotyped for each locus.

b A_N_ = Number of alleles observed.

c H_o_ = Observed heterozygosity.

d H_e_ = Expected heterozygosity.

e F_is_ = Inbreeding coefficient.

Estimates of H_e_ for historical populations of ocelot in Texas were similar to those observed in Los Ebanos, Mexico (Tables 4 and 5). Cameron had significantly lower H_e_ (*P*<0.05). In the Texas historical sample, H_e_ was also significantly higher than in Willacy (*P*>0.05). With only a mean of 5 samples per locus, the sample size for the historical population was not high enough to allow rigorous tests for genetic divergence. However, pair-wise F_st_ was estimated between each population to examine the consistency of the microsatellite data set with the mtDNA data. The most divergent populations were Cameron and historical Texas, with F_st_ = 0.367 (*P*<0.001), and the lowest divergence was between Willacy and Los Ebanos with F_st_ = 0.146 (*P*<0.001) (Table 6).

**Table 6:** Pairwise F_st_ values estimated from microsatellite data for 3 contemporary ocelot populations in Texas (Cameron and Willacy) and Mexico (Los Ebanos) and ocelot museum specimens collected in that region (Texas Historical).

	Cameron	Willacy	Los Ebanos,Mexico	TexasHistorical
Cameron	–	<0.001	<0.001	<0.001
Willacy	0.181	–	<0.001	<0.001
Los Ebanos,Mexico	0.330	0.146	–	<0.001
Texas Historical	0.367	0.241	0.186	–

The *P*-values based on 9,999 permutations are above the diagonal.

### Change in Diversity from Mid-1980s to 2005

**Figure 3:**
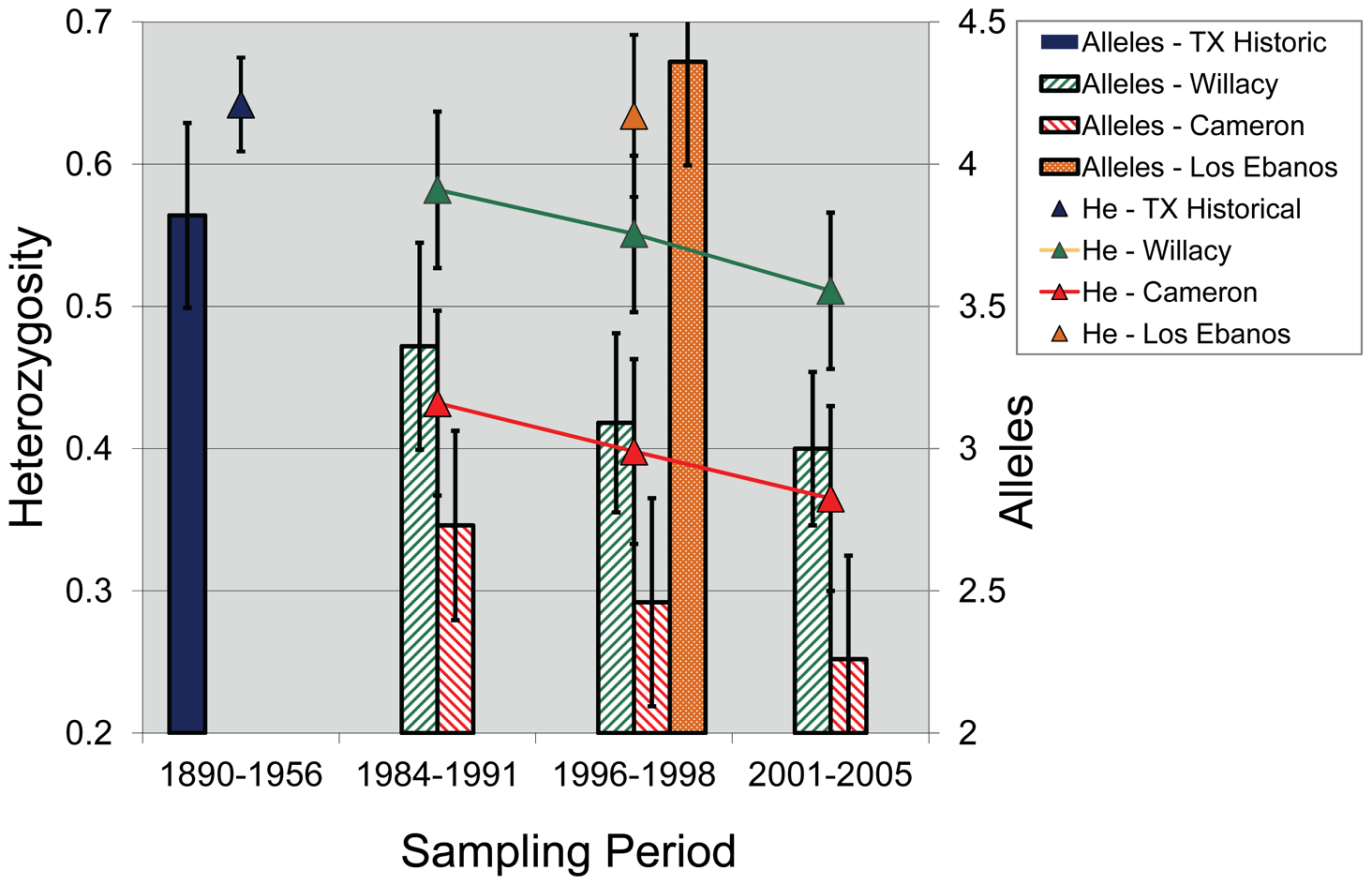
Temporal changes in microsatellite diversity of 11 loci within ocelot populations in Texas and northeastern Mexico. Willacy and Cameron are counties in Texas and Los Ebanos is a ranch in Mexico. Historical ocelot populations were sampled using museum specimens that originated in the Texas region.

We also examined temporal microsatellite variation between 1986 and 2005 in the two remaining Texas populations. Samples from each of the areas were separated into 3 temporal groups: Cameron (1) 1986–1989, n = 10, (2) 1996–1998, n = 10, and (3) 2001–2005, *n* = 10 and Willacy (1) 1984–1991, n = 8, (2) 1996–1998, n = 10, and (3) 2001–2005, n = 10. There was a reduction in both allele number and H_e_ in each of the populations (Figure 3). Cameron initially had H_e_ = 0.432 and A_N_ = 2.73, but in more recent samples the estimates dropped to H_e_ = 0.365 and A_N_ = 2.36. The Willacy population showed a similar pattern, with initial H_e_ = 0.582 and A_N_ = 3.36 reducing to H_e_ = 0.511 and A_N_ = 3.00. During each respective period, variation in Willacy was higher than in Cameron.

## Discussion

Comparisons of contemporary ocelot populations to historical specimens provided strong evidence for a loss of genetic diversity in Texas over the last 100 years. The historical Texas population examined had haplotype and nucleotide diversity comparable to a contemporary population in northern Mexico, and substantially higher than what remains in Texas. Two of the haplotypes observed in museum specimens were no longer detected among extant ocelots. By the 1980s, the federally protected Cameron population at Laguna Atascosa National Wildlife Refuge lost all variability at the mtDNA control region sequenced in this study. The population on private lands in Willacy County still retained 2 haplotypes through mid-1990s; however, by 2005 it was also fixed for the haplotype present in Cameron. These observations are consistent with the small effective population size previously estimated in Texas circa 1995 (N_e_<14 in Cameron and N_e_<4 in Willacy) [12] and complete population isolation [11]. In contrast, ocelots in northeastern Mexico retained historical levels of mtDNA diversity through 2004, suggesting larger ocelot populations south of the United States.

The mtDNA haplotype no longer detected in either the Willacy or Cameron populations was last observed in a road-killed ocelot near Port Mansfield in 2004. This suggests that there may be habitat in southern Texas adjacent to the known populations harboring ocelots with more variation. The Willacy population resides on the western and southern edge of large ranches that have retained more native thornshrub habitat than areas surrounding the Laguna Atascosa National Wildlife Refuge in Cameron County. These private lands are critical for ocelot recovery in the United States.

The microsatellites examined also depict genetic erosion in Texas during the 20^th^ century. Genotyping of museum samples presents difficulties because of the degradation and low quantity of DNA extracted from some specimens [56]. We unfortunately had poor success, and as a consequence, the number of loci scored in historical samples was low. Nevertheless, despite having genotypes for a mean of only 5 ocelot specimens per locus, genetic diversity was historically higher than in contemporary Texas populations. A substantial number of alleles have disappeared. As with mtDNA haplotypes, the remnant Texas populations continued to lose microsatellite diversity through 2005. Cameron exhibited an 18% reduction in H_e_ and Willacy had a 14% reduction. This further corroborates that ocelots in Texas continue to experience genetic drift and inbreeding as a result of small effective population size and isolation [12].

Lower levels of neutral genetic diversity have been observed in peripheral populations subjected to island effects during range contractions and expansions [57], [58] and for mammal populations at higher latitudes [59]. The lower genetic diversity observed previously in Texas ocelot populations [11], [12], [43] could have been the result of these evolutionary processes. Indeed, in the two ocelot phylogeographic studies based on mtDNA, variation in South America was higher than in northern latitudes [43], [60]. However, the greater mtDNA and microsatellite diversity we observed in specimens collected between 1853 and 1956, along with the continued decline in the remnant populations through the 1990s, strongly support the idea that genetic diversity was reduced by the extensive human induced population decline during the 20^th^ century. It is likely that both of these processes have affected ocelot diversity in the United States. A range-wide study comparing the core and peripheral parts of the ocelot distribution using nuclear markers would provide additional information regarding the extent to which Pleistocene fluctuations in northern latitudes contributed to lower genetic diversity in Texas.

Human development and habitat modifications were extensive during the 20^th^ century in the Lower Rio Grande Valley [61]. Large tracts of dense thornshrub communities preferred by ocelots were eliminated to accommodate agriculture, grazing of cattle, and housing developments [3], [61], resulting in patches of isolated habitat. Recent loss of habitat and increased isolation of ocelot populations in Texas seem to be the major proximate causes of the reduction in genetic variation. Similar anthropogenic effects have been observed in diverse taxa including Amur leopards (*Panthera pardus orientalis*) [62], Florida panthers (*Puma concolor coryi*) [63], European otters (*Lutra lutra*) [64], greater prairie-chickens (*Tympanuchus cupido pinnatus*) [38], heath hens (*Tympanuchus cupido cupido*) [32], black-footed ferrets (*Mustela nigripes*) [65], grey wolves (*Canis lupus*) [66], and arctic foxes (*Alopex lagopus*) [67].

The rapid loss of genetic diversity observed in these species may influence their future persistence. Inbreeding depression in small populations has been found to negatively affect many traits including larval growth rates in the natterjack toad (*Bufo calamita*) [68], adult growth rate, development time and fecundity in *Daphnia* [69], fecundity and survival in the Glanville fritillary butterfly (*Melitaea cinxia*) [27], fecundity in wolf spiders (*Rabidosa punctulata* and *Rabidosa rabida*) [70], and fecundity, body size, and survival of many mammals including chimpanzees (*Pan troglodytes*) [71], tigers (*Panthera tigris*) [72], harbor seals (*Phoca vitulina*) [36], and Florida panthers (*Puma concolor*) [37]. Isolation of the remaining ocelot populations is becoming more severe; the Lower Rio Grande Valley is one of the fastest growing regions of the United States [3], [73]. These trends will likely result in continued small effective population size and isolation, which will foster additional loss of variation through genetic drift and increased homozygosity. As previous studies on small populations have documented many negative fitness consequences [22], [28], [63], [71], [74]–[76], the remaining ocelots in Texas are also expected to experience reductions in reproductive potential and a higher susceptibility to factors threatening their long-term survival (e.g., disease and road mortality).

One short-term management strategy for increasing the probability of ocelot persistence in the United States is to restore some of the genetic diversity that was lost during the 20^th^ century and reduce inbreeding by introducing unrelated individuals. Even one migrant per generation may alleviate negative effects associated with inbreeding depression and loss of adaptive variation [37], [77]. The positive influence of restoring gene flow with an influx of individuals from different populations has been supported by observations across diverse wild species [37], [78], [79]. Therefore, it would be beneficial to initiate recovery strategies that would avert the threat of inbreeding depression and loss of adaptive variation before it is phenotypically manifested in the remaining Texas ocelots.

Translocation of ocelots from either more diverse source populations (e.g., northeastern Mexico), or reciprocally between the two remnant Texas populations, is a practical method for restoring a portion of the historical genetic diversity. This is the most feasible immediate strategy for combating isolation, inbreeding, and genetic drift because landscape restoration of thornshrub corridors required to increase natural connectivity is predicted to take a long time (i.e. >20 years) [80]. Due to the extensive development along the lower stretches of the Rio Grande, connectivity with the northeastern Mexico populations cannot be established in the foreseeable future. In addition, an increase in suitable habitat by itself is not always correlated with improved fitness and sustainability of genetically depauperate populations [78], [79]. In a habitat-based population viability analysis model, partnering the augmentation of Texas populations via translocations with the reduction of road mortality by constructing culverts was an effective short-term strategy for decreasing ocelot extinction probability [10].

Northeastern Mexico is the most appropriate source of individuals for translocations to Texas based on the higher genetic diversity, ecological similarity between these localities, and their close phylogenetic relationship [11], [43]. A previous mtDNA study found parts of Central America also appear to be in the same Evolutionary Significant Unit (ESU) as Texas and Mexico, despite being currently classified as different subspecies [43]. Therefore, if ocelots from Mexico are not available for translocations, then other potential sources could include Central American countries in this ESU, such as Belize. However, this phylogeographic analysis was based on mtDNA; therefore, the ocelot ESU designations need to be corroborated with nuclear markers.

Reciprocal translocations between the Willacy and Cameron populations should be implemented immediately because this would mimic natural connectivity, reduce inbreeding and drift, and restore a substantial amount of historical variation, while being financially, logistically, and politically more feasible than translocations from foreign countries. The endangered ocelot is the last Neotropical felid that still has viable breeding populations in the United States. Management strategies should incorporate restoration of genetic diversity and population connectivity to ensure persistence and expansion of these remnant populations in order to achieve recovery of the ocelot in its northernmost range country.

## Supporting Information

Table S1 **Information on the ocelot museum specimens sampled during this study.**
(XLSX)
